# Biological Characterization of Ti6Al4V Additively Manufactured Surfaces: Comparison Between Ultrashort Laser Texturing and Conventional Post‐Processing

**DOI:** 10.1002/adhm.202402873

**Published:** 2024-10-22

**Authors:** Maria Sartori, Chiara Bregoli, Melania Carniato, Luca Cavazza, Melania Maglio, Gianluca Giavaresi, Carlo Alberto Biffi, Jacopo Fiocchi, Emanuele Gruppioni, Ausonio Tuissi, Milena Fini

**Affiliations:** ^1^ Surgical Sciences and Technologies IRCCS Istituto Ortopedico Rizzoli Via Di Barbiano, 1/10 Bologna 40136 Italy; ^2^ Institute of Condensed Matter Chemistry and Technologies for Energy (ICMATE) (Consiglio Nazionale delle Ricerche – CNR) Via Gaetano Previati, 1/E Lecco 23900 Italy; ^3^ INAIL Centro Protesi Via Rabuina 14, Vigorso di Budrio Bologna 40054 Italy; ^4^ Scientific Direction IRCCS Istituto Ortopedico Rizzoli Via Di Barbiano, 1/10 Bologna 40136 Italy

**Keywords:** bioactivity, cytotoxicity, laser texturing, LPBF Ti6Al4V ELI, surface finishing

## Abstract

Among Additive Manufacturing (AM) technologies, Laser Powder Bed Fusion (LPBF) has made a great contribution to optimizing the production of customized implant materials. However, the design of the ideal surface topography, capable of exerting the best biological effect without drawbacks, is still a subject of study. The aim of the present study is to topographically and biologically characterize AM‐produced Ti6Al4V ELI (Extra Low Interstitial) samples by comparing different surface finishing. Vertically and horizontally samples are realized by LPBF with four surface finishing conditions (as‐built, corundum‐sandblasted, zirconia‐sandblasted, femtosecond laser textured). Bioactivity in vitro tests are performed with human osteoblasts evaluating morphology, metabolic activity, and differentiation capabilities in direct contact with surfaces. Scanning electron microscope and profilometry analysis are used to evaluate surface morphology and samples’ roughness with and without cells. All tested surfaces show good biocompatibility. The influence of material surface features is evident in the early evaluation, with the most promising results of morphological study for laser texturing. Deposition orientations seem to influence metabolic activities, with XZ orientation more effective than XY. Current data provide the first positive feedback on the biocompatibility of laser texturing finishing, still poorly described in the literature, and support the future clinical development of devices produced with a combination of LPBF and different finishing treatments.

## Graphical abstract

This work aims to provide a topographical and biological characterization of Additively Manufactured (AMed) Ti6Al4V ELI surfaces subjected to different surface finishing techniques. Unlike traditional biomedical sandblasting methods, femtosecond laser texturing generates new surface roughness and morphologies, which show promise for enhancing the differentiation and proliferation of human bone cells. This work lays the groundwork for optimizing the surfaces of AMed medical devices.

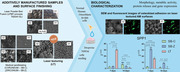

## 1 Introduction

Titanium and Ti‐based alloys are well‐established and represent one of the mostly materials for medical implants.^[^ ^1^ ^]^ Their biocompatibility, chemical inertness, and mechanical properties made them the gold standard for biomaterials, boasting an extensive array of biomedical applications, especially in the orthopedic field. In the latter, a close and active interaction between human tissues and biomaterial is crucial to ensure stable and prolonged osteointegration. Within this scenario, the surface characteristics of the materials – such as topography, chemistry, wettability, and mechanical properties – obtained with several modification techniques play a pivotal role.^[^ ^2^ ^]^ They serve as a foundation for the on‐going cross‐talk and interplay between tissue elements and implanted materials both in the initial phase of interaction and in influencing the establishment of long‐lasting connections.^[^ ^3^ ^]^

In mean time, Additive Manufacturing (AM) technology has become increasingly widespread in the medical field.^[^ ^4^, ^5^, ^6^, ^7^ ^]^ AM design freedom permits the realization of complex structures, e.g. lattice structure, and the production of personalized, patient‐matched devices, able to address patient‐specific needs.^[^ ^8^, ^9^ ^]^ Among AM processes, Laser Powder Bed Fusion (LPBF) is the most adopted in the medical field.

In this respect, LPBF Ti6Al4V alloy is known to present excellent biocompatibility, as shown by numerous cytotoxicity and bioactivity tests available in the literature.^[^ ^8^, ^10^, ^11^ ^]^ The LPBF surface was also demonstrated to present a typical roughness, which is advantageous for osteointegration^[^ ^12^ ^]^: Suresh at al.^[^ ^8^ ^]^ observed enhanced adhesion and proliferation of bone cells in contact with both porous and bulk LPBF Ti6Al4V samples. Likewise, an optimal proliferation of fibroblasts on LPBF Ti6Al4V surfaces was recorded.^[^ ^13^ ^]^

Nevertheless, the roughness resulting from LPBF, which was demonstrated to be beneficial for osteointegration, presents some drawbacks, too: particles partially adhering to the surface could be detached and activate a negative immune response.^[^ ^14^ ^]^ Moreover, poor surface finishing may lead to stress concentration sites, which negatively affect static and fatigue mechanical behavior.^[^ ^14^ ^]^

Therefore, a lot of effort is being put into finding the best post‐processing method for LPBFed parts: ideally, excessive roughness and partially adhering particles should be removed, while maintaining the conductivity provided by the rough surface, as it is well established that surface topography plays a crucial role in influencing biocompatibility.^[^ ^15^ ^]^ The most established post‐processing techniques include mechanical polishing, chemical passivation, chemical etching, and surface treatments with abrasive media, such as sandblasting.^[^ ^14^, ^16^, ^17^, ^18^ ^]^ Bernhardt et al.^[^ ^19^ ^]^ investigated the effect of vibratory surface finishing, electro‐polishing, and plasma polishing on LPBFed Ti6Al4V samples confirming their excellent biocompatibility and their fitness for biomedical applications. Sandblasting with zirconia or corundum media is widely adopted in the medical field, too.^[^ ^20^, ^21^, ^22^, ^23^ ^]^ Nevertheless, these methods allow no tailoring in surface finishing. In this respect, laser texturing was demonstrated as a promising route for tailoring surface properties^[^ ^14^, ^24^ ^]^: ultrafast lasers, i.e., those based on picosecond, femtosecond, and nanosecond pulses, acquired popularity thanks to their ability to induce strictly controlled morphological features with different groove depth and topography.^[^ ^14^, ^15^, ^25^, ^26^ ^]^ Cell and tissue responses are influenced by surface changes: morphological modifications can influence in a pivotal manner cell adhesion and colonization as well and changes in surface topography may, in turn, affect chemical‐physical properties, such as wettability or functional group exposition, which strongly affect the bone cell material interaction cascade.^[^ ^27^ ^]^ Chen et al.^[^ ^27^ ^]^ investigated the morphology and adhesion of osteosarcoma cells on polished, zirconia‐blasted, and laser‐textured Ti6Al4V samples produced by conventional processes: the different micro‐scale topological features strongly affected the cell adhesion. Laser micro‐grooved geometries with depth of 10 µm and spacing of 20 µm mostly influenced the cell's orientation and adhesion. Micrometric and nanometric linear and pattern grooves were demonstrated to guide cellular orientation.^[^ ^26^, ^28^, ^29^ ^]^ Branemark et al. developed an improved bone‐implant surface anchorage with site‐specific laser‐induced micro‐ and nano‐scale grooves: the implant performed well in rabbit tibia, demonstrating the benefits of surface oxide induced by laser treatment, too.^[^ ^30^ ^]^ Pfleging et al.^[^ ^15^ ^]^ investigated the possibility of obtaining linear and dimple geometries by nanosecond laser texturing: the increase in surface area leads to an increase in wettability with consequent benefits on cell attachment. Although cell bioactivity and wear resistance were improved by nano‐laser texturing, cell density decreased close to the dimple‐textured surface.^[^ ^26^ ^]^ Worts et al.^[^ ^31^ ^]^ investigated the morphology induced by femtosecond laser on LPBFed Ti6Al4V samples: nevertheless, no biological characterization was reported.

The relevance of studying innovative surface finishing processes, able to address the drawbacks related to LPBF is therefore evident; surface finishing techniques able to tailor the surface topology need to be better established and researched.

In this respect, laser texturing represents one of the most innovative and promising surface finishing techniques for tailoring the surface of biomedical alloys and thus the cell's response.

The current research aims to comprehensively explore the effects of different surface finishing techniques on in vitro biological responses of bone cells. In this scenario of rapid materials development and improvement also driven by LPBF advent, thorough investigations are necessary to determine how the combination of AM and surface finishing techniques effectively affects cellular behaviors such as morphogenesis, proliferation, and differentiation. Indeed, the LPBF technique results in a highly different surface depending on the orientation of the part relative to the building platform. To achieve that vertically and horizontally produced Ti6Al4V ELI (Extra Low Interstitial) samples were realized by means of LPBF and studied in four different surface finishing conditions: as‐built, sandblasted with corundum and zirconia, and textured by femtosecond laser (fs‐laser). Optimized fs‐laser texturing parameters were chosen. Surface characterizations were carried out by means of Scanning Electron Microscope (SEM) observation, 3D profilometry analysis, and wettability quantification. In vitro biological investigations were performed using human primary osteoblasts to quantitatively asses, for the first time, the effect of these different surface finishing conditions combined with AD materials on osteoblast cell performance. The study focused on adhesion, proliferation, and differentiation in order to gather new information about these new classes of materials and further optimize their performance.

The present work aims to exploit the use of deep laser texturing as post‐processing of Ti6Al4V ELI AMed parts, fabricated by the LPBF process which, to date despite its promising impact on surface properties in the biomedical field, is still poorly addressed in the literature. The morphology and biological performances of the laser‐textured surfaces were compared with conventionally finished surfaces, already implemented in the clinical field. The use of laser texturing as post‐processing of AMed parts paves a promising way in tailoring and enhancing the local morphology of the finished surfaces for promoting different biological.

## 2 Experimental Section

### 2.1 Sample Preparation

**Figure 1:**
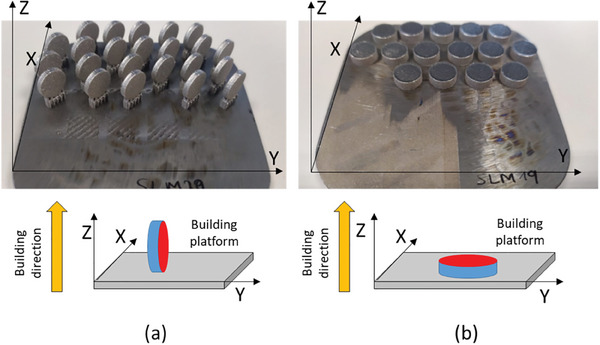
Schematic representation of discs fabricated in different building orientations. a) XZ samples fabricated in the vertical direction; b) XY samples fabricated in the horizontal direction.

Disc‐shaped samples (10 mm in diameter, 2 mm in thickness) were produced by LPBF (mod. AM400 by Renishaw), starting from gas‐atomised, medical‐grade Ti6Al4V ELI powder. The used processing parameters, which had been optimized in previous work,^[^ ^32^ ^]^ allowed to achieve relative densities in excess of 99.7%. Samples were produced with their diameter oriented either parallel (XY) or perpendicular (XZ) to the building direction, so as to study the effect of different surface orientations on the behavior of the material^[^ ^33^ ^]^ (**Figure** **1**).

All specimens were subjected to a heat treatment to remove residual stresses and allow the desired microstructural modifications: samples were held at 850 °C for 1 h under high vacuum and then naturally cooled to room temperature.

### 2.2 Laser and Conventional Surface Finishing

Three types of surface finishing were performed starting from the as‐built (AB) surface condition: ultrashort laser texturing (namely LT), representative of an advanced process, and two conventional ones, sand blasted with corundum (SB‐C) and sand blasted with zirconia (SB‐Z) particles (Al Ti Color srl, Padova, Italy). The conventional post‐processing was carried out with standard biomedical industrial parameters in order to compare the industrial reference with the advanced post‐processing method performed via ultra‐short laser texturing. The sandblasting technique consists of a first cleaning of the samples, a manual sandblasting of each specimen, and a final cleaning of the sample to remove any residual of the sandblasting procedure. Surface finishing conditions strongly affect the roughness and morphology of the surface with a consequent huge impact on cellular interaction.^[^ ^34^ ^]^

**Figure 2:**
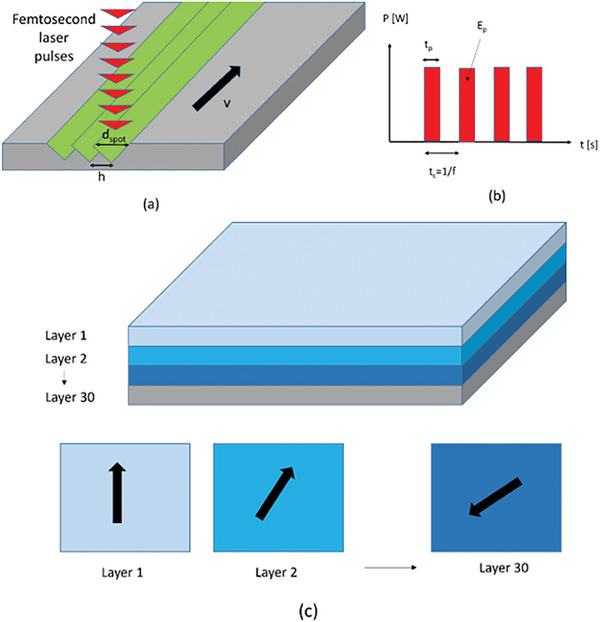
Schematic of the principal spatial (a) and temporal (b) parameters adopted during laser texturing, as well as the orientation change of the laser passes on different levels (c).

Laser texturing was performed by means of a femtosecond (fs) laser (mod. carbide 40 W from Light Conversion); the main characteristics of the laser equipment are listed in **Table** **1**. The laser processing was carried out by scanning the surface of LPBFed discs without any protective atmosphere. Schematic of the laser texturing process is depicted in **Figure** **2**; in detail, Figure 2a shows the scanning strategy adopted on the single layer, where the beam size (d_spot_) and the hact distance (h) can be clearly seen. Then, Figure 2b shows the temporal profile of the pulsed wave emission, showing the pulse duration (t_p_) and the corresponding frequency (f).

**Table 1:** Main characteristics of the femtosecond laser equipment.

Parameters	Value
Power [W]	40
Emission wavelength [nm]	1030
Frequency range [kHz]	100‐1000
Laser spot size [µs]	16
Polarization	circular

A linear scanning strategy was carried out, tilting the laser direction of 67° passing from one layer to the next one; a total number of 30 laser passes were adopted for multiple scanning of the surface (see Figure 2c). Starting from previous exploratory work,^[^ ^24^ ^]^ the authors selected the process parameters for performing a deeper ablation for processing the AMed surfaces, which are typically irregular and characterized by high roughness values, for fabricating a smoother surface with tailored micro‐structures, which may be promising for biological aspects.

Therefore, the principal process conditions explored in the work,^[^ ^24^ ^]^ characterized by energy enough to promote surface modification during the laser scanning on a single layer, were oriented to the use of lower energy (lower power, higher scanning speed) to be implemented for scanning the surface several times (up to 30 passes overlapped each others).

The adopted process parameters in the present work are listed in **Table** **2**.

**Table 2:** Process parameters adopted for laser texturing of Ti6Al4V ELI asbuilt discs.

Parameters	Value
Power [W]	10
Pulse duration [fs]	100
Frequency range [kHz]	400
Velocity [mm s^−1^]	2500
Hatch/point distance [µm]	6.25
Number of passes [‐]	30
Rotation angle [deg]	67
Pattern	Linear

### 2.3 Sample Topography Characterisation

The surface morphology of the produced samples, in all the considered conditions, was characterized by means of Scanning Electron Microscopy (SEM, mod.Leo 430 by Zeiss, operating at 20 KV) and 3D non‐contact confocal profilometry (Sensofar S‐neox, vertical resolution of 1.5 nm). The surface roughness (Sa) was quantitatively measured by collecting measurements on different areas (200 × 297 µm^2^) of the observed samples.

### 2.4 Cell Culture and Seeding Procedure

Before carrying out the in vitro tests, all samples underwent steam sterilization in an autoclave (Getinge Disinfectation AB‐HS33 1p). Normal human osteoblast cells (NHOst LOCC2538; LONZA, Verviers, Belgium) were utilized to evaluate biocompatibility as they are primary cells directly derived from human bone tissue and retain the physiological properties of their tissue of origin. This ensures reliable, reproducible, and high‐quality results that closely mimic in vivo responses. The cells were cultivated and expanded in osteoblast basal medium (OBM Osteoblast Growth and Differentiation Basal Medium; LONZA), supplemented with the appropriate additives (OGM Osteoblasts Growth SingleQuots kit, LONZA), 10% fetal bovine serum (FBS, EUROCLONE, Pero, Milano, Italy), 100 U mL^−1^ penicillin, 100 µg mL^−1^ streptomycin, (SIGMA, St. Louis, MO) under standard conditions (37 °C, 5%CO_2_/95%air, humidified atmosphere).

**Figure 3:**
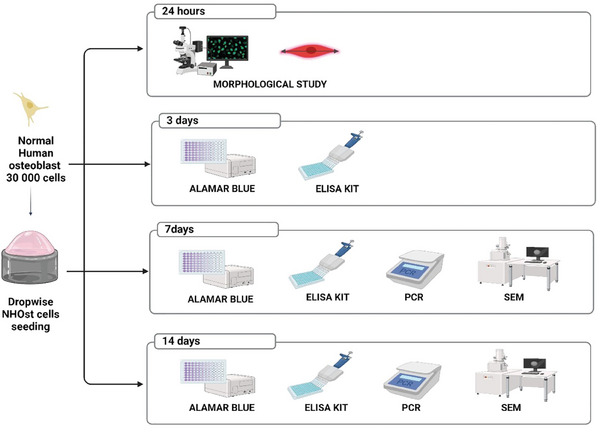
Biocompatibility study “work‐plan”. Created with BioRender.com.

Cells were detached using tripsin/EDTA (Sigma Aldrich, Missouri, USA) for 5 min and counted in a Neubauer chamber using Erythrosin B exclusion dye (Thermo Fisher, Kandel, Germany). Subsequently, cells were resuspended and seeded at the concentration of 8×10^2^ per material surface for morphological study at 24 h and 3 × 10^4^ cells per material surface for metabolic activity, gene expression, and SEM analyses as detailed in **Figure** **3**. The cells were utilized in passage 4.

For seeding, a small volume (85 µL) was added dropwise to ensure the complete coverage of the samples. Then, NHOst cells were left on the materials for 1 h at 37 °C without an additional medium to facilitate cell adhesion to their surfaces. Subsequently, the medium (OBM Osteoblast Growth and Differentiation Basal Medium) was added to completely cover the substrates. Twenty‐four hours after seeding, the samples were transferred from the original seeding wells to a new plate, and the following experimental conditions were established:

– XY – XZ SB‐C;
– XY – XZ SB‐Z;
– XY – XZ LT.

NHOst seeded onto polystyrene or glass slides were adopted as 2D controls of the culture for the osteoblast morphology and differentiation capability maintenance. The cultures were kept at 37 °C in a 5% CO_2_ humidified atmosphere for 3, 7, and 14 days.

### 2.5 Morphological Study at 24 H

Early morphological features of NHOst onto experimental and control samples were visualized by staining the cells at 24 h of culture. The medium was aspirated, and the samples were washed with Phosphate Buffered Solution 1X (PBS, Gibco, Thermo Fisher). Then, they were fixed with a cold solution of 3.7% paraformaldehyde solution in PBS for 30 min, permeabilized using 0.5% Triton X‐100 for 10 min, and subsequently blocked with a solution containing 1% of bovine serum albumin (BSA) in PBS for 30 min. To visualize the actin filaments of the cytoskeleton, incubation with FITC‐conjugate phalloidin solution (50 µg mL^−1^) (Sigma–Aldrich, catalog number: P5282) in PBS was performed at 37 °C for 45 min. Finally, samples were incubated with the specific nuclear dye DAPI solution (2 µg mL^−1^) (Sigma–Aldrich, catalog number: D8417) for 10 min in the dark and washed twice with PBS. After this procedure, they were stored at 4 °C until image acquisition.

The visualizations of the stains were carried out using an inverted microscope equipped with an epifluorescence configuration (Eclipse TiU, NIKON Europe BV, NITAL SpA, Milano, Italy). Multiple fields were captured for each substrate at 20× and 40× magnifications. Within each field, individual cells were delineated to perform specific morphometric measurements. Area, perimeter, circularity, roundness, and aspect ratio were determined using ImageJ software (Version 1.53c, National Institutes of Health, Bethesda, MD, USA). Circularity was computed as

(1) $$Circularity=4\pi \cdot \frac{cell\;area}{cell\;perimeter^{2}}$$

roundness was defined as

(2) $$Roudness=\frac{4}{\pi }\cdot \frac{cell\;area}{major\;axis^{2}}$$

and aspect ratio was calculated as

(3) $$Aspect\;ratio=\frac{major\;axis}{minor\;axis}$$

### 2.6 Cell Metabolic Activity

Alamar Blue test was employed to investigate cellular metabolic activity (Alamar Blue, Serotec, Oxford, UK). The dye was added to both experimental and control conditions in a 1:10 v/v at 3, 7, and 14 days, in accordance with the manufacturer's guidelines. The reagent was then incubated with experimental and control samples and conditions at 37 °C in a 5% CO_2_ humidified atmosphere for 4 h. The Alamar Blue assay operates on the principle of utilizing an oxidation‐reduction indicator that changes color in response to the chemical reductions induced by the active cell mitochondria. The resulting fluorescence was measured at 530ex–590em nm wavelengths with a Micro Plate Reader (VICTOR X2030, Perkin Elmer, Milano, Italy) and was expressed as Relative Fluorescence Units (RFUs). A culture medium containing the reagent but devoid of cells was utilized as a control for background fluorescence.

### 2.7 Gene Expression

Gene expression analyses were performed at 7 and 14 days of culture. Total RNA was extracted using the PureLink RNA Mini Kit (Ambion by Life Technologies, Carlsbad, CA, USA) and the extracted RNA was quantified using a spectrophotometer (NANODROP 2720, Applied Biosystem, Waltham, MA, USA). Afterward, the RNA was reverse transcribed with the Superscript Vilo cDNA synthesis kit (Life Technologies, Carlsbad, CA, USA) and subsequently diluted to a final concentration of 5 ng/µL. Gene expression analysis was performed through semi‐quantitative PCR, employing the SYBR Green PCR master mix (QIAGEN GmbH, Hilden, Germany), and conducted on a LightCycler 2.0 Instrument (Roche Diagnostics, Basel, Switzerland; GmbH, Hilden, Germany; Manheim, Atlanta, GA, USA) adopting gene‐specific primers reported in **Table** **3**. The multistep procedure protocol included an initial denaturation cycle at 95 °C for 15 min, followed by 25–40 cycles of amplification, and a subsequent melting curve analysis to assess the specificity of the amplicons. The mean threshold cycle was utilized to obtain relative expression, employing the Livak method (2^−ΔCt^), with GAPDH as the reference gene. Each sample was assayed in triplicate.

**Table 3:** Details of primers used for gene expression analysis.

Gene	Primer forward (5′→3′)	Primer Reverse (5′→3′)	Amplicon Lenght	Annealing Temperature
*GAPDH*	QuantiTect Primer Assay (Qiagen) Hs_GAPDH_1_SG		95 bp	55 °C
*ALPL*	QuantiTect Primer Assay (Qiagen) Hs_ALPL_1_SG		110 bp	55 °C
*COL1A1*	QuantiTect Primer Assay (Qiagen) Hs_COL1A1_1_SG		118 bp	55 °C
*BMP2*	TTTGACCAGAGTTTTTCCATG	GAAGCAGCAACGCTAGAAGA	130 bp	60 °C
*SPP1*	QuantiTect Primer Assay (Qiagen) Hs_SPP1_1_SG		115 bp	55 °C
*TGFbeta*	GTTCAGGTACCGCTTCTCG	CCGACTACTACGCCAAGGA	138 bp	60 °C

### 2.8 Immunoenzymatic Assays

After 3, 7, and 14 days of culture, supernatants from each condition were harvested, centrifuged to remove particulates, and stored at −80 °C until use. Enzyme‐linked immunosorbent assays (ELISA) were performed to quantitatively measure the release of crucial cytokines and main osteogenic markers. Briefly, 100 µL of cell culture supernatant was added to 96 well plates coated with antibody specific to human Osteonectin, also known as “secreted protein acidic and rich in cysteine” (hSPARC, Code: EK‐1210 provided by Boster Bio (Pleasanton, CA, USA), Collagen type I (Coll‐I; Catalog: SEA571Hu), Interleukin‐1 beta (hIL‐1β, Catalog: SEA563Hu) and Interleukin‐6 (hIL‐6, Catalog: SEA079Hu) all of which were supplied by Cloud‐Clone Corp. (USA). Additionally, Osteocalcin (h‐Osteocalcin, Catalog: BMS2020INST) supplied by Invitrogen, was also performed. For each protein, the analysis was performed following the manufacturer's provided guidelines. The concentration of the markers was calculated by reading the absorbance at 450 nm on a spectrophotometer (Micro Plate reader – Bio‐Rad Laboratories, CA). Each sample underwent quadruplicate testing, except for the cellular control, which was assessed in triplicate.

### 2.9 Scanning Electron Microscopy

NHOst seeded onto different substrates were also examined using Scanning Electron Microscopy (SEM) at 7 and 14 days. At the end of each experimental time, materials were fixed with 2.5% glutaraldehyde in 0.1 m phosphate buffer (pH 7.4) for 1 h. After that, materials were dehydrated using a series of graded ethanol solutions (30%, 50%, 70%, and 95%) for 15 min each and ethanol 100% for 1 h. After two consecutive passages in hexamethyldisilazane (5 min each), the samples were left to dry overnight (Merck KGaA, Darmstadt, Germany)

The surface morphology of the produced samples with and without seeded cells, in all the considered conditions, was characterized by means of SEM (SEM, mod.Leo 430 by Zeiss, operating at 20 KV) Average surface roughness in the samples before proceeding with cell seeding was measured and compared.

### 2.10 Statistical Analysis

All statistical analyses were performed with SPSS v.19.0 (IBM Corp., Armonk, NY, USA) and graphs were created with GraphPad Prism (Version 9, GraphPad Software, la Jolla, USA). Quantitative data are presented as mean and 95 Confidence Intervals; categorical data as percentages. The Shapiro test was used to assess the normality of the distributions. The Generalized Linear Mixed Models analysis with Morphometric descriptors, metabolic activity, Gene Expression, and Immunoenzymatic assays as dependent variables, surface orientation, surface finishing conditions, and follow‐up times as fixed effects were used to assess the influence of material surface parameters on the dynamic of the aspects of interest. The chosen distribution and link function were determined according to the distribution of the parameters: so far we used gamma distribution with log‐link function for strongly asymmetrical and non‐normally distributed variables and normal distribution with log‐link function for asymmetrical log‐normally distributed variables. The pairwise multiple comparisons were carried out with Sidak test correction. The level of statistical significance was set at *p* < 0.05.

## 3 Results and Discussion

### 3.1 Surface Characterization

**Figure 4:**
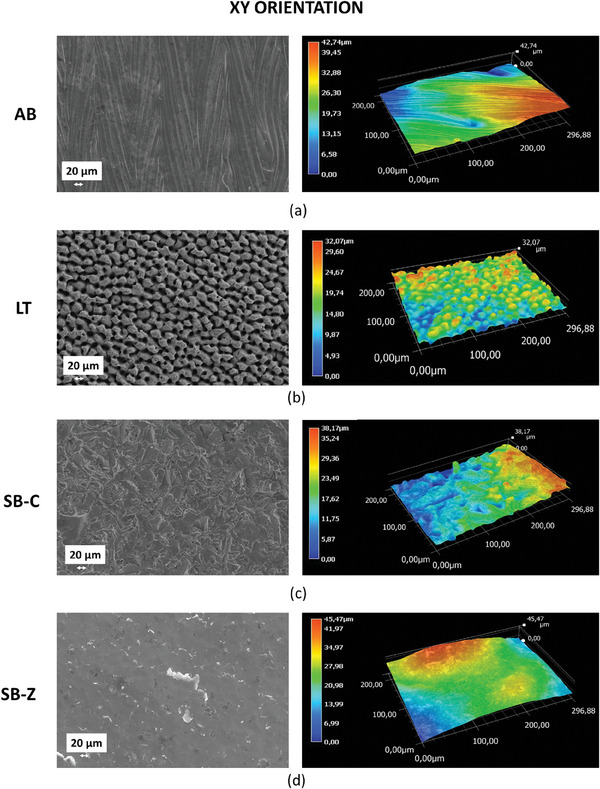
SEM images and 3D profilometries of surfaces under different finishing conditions: a) as‐built XY; b) laser‐textured XY; c) corundum‐sandblasted XY; d) zirconia‐sandblasted XY.

**Figure 5:**
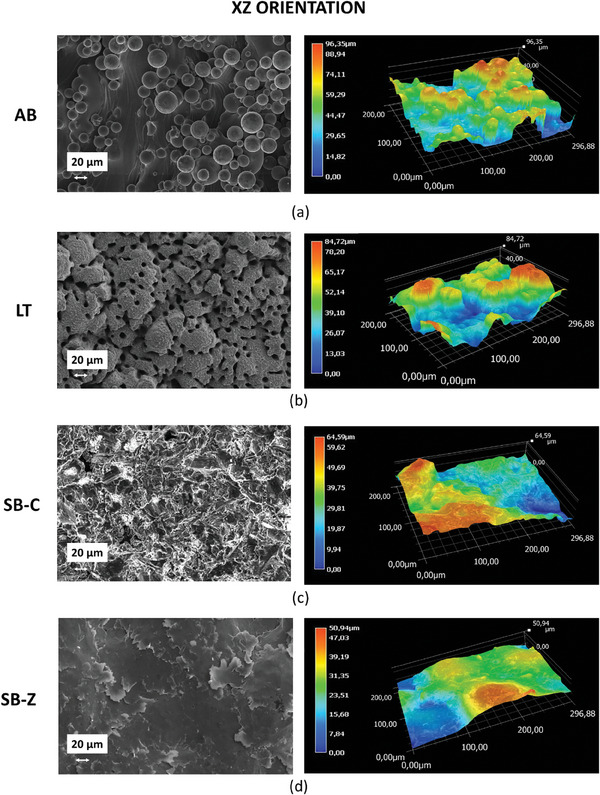
SEM images and 3D profilometries of surfaces under different finishing conditions: a) as‐built XZ; b) laser‐textured XZ; c) corundum‐sandblasted XZ; d) zirconia‐sandblasted XZ.

AB surfaces, lying in the XY and XZ planes, exhibit the typical morphologies from the LPBF process (**Figures** **4** and **5**). In the XY view, striations left by the laser scans can be easily observed. On the contrary, the XZ view shows the presence of unmelted powder, partially adhering to the vertical walls, and giving rise to an irregular surface.

The femtosecond laser texturing carried out with a considerable number of overlapping laser scans, heavily modified the original AB surfaces, producing closely packed micro‐pillars on both orientations. Profilometry reveals that on the XY surface, such pillars are ≈14 µm wide and 16 µm high, while the spacing setting them apart is ≈15 µm. The original waviness of the AB condition was completely deleted by the laser texturing process: in fact, there are no traces of the scans promoted during the LPBF process, and only limited local depressions, lying beneath the mentioned pillars, can be observed from time to time. Laser texturing promotes the formation of similar pillars on the vertical surfaces of the LPBF samples, as well, and only limited traces of unmelted particles from the LPBF process are left behind. Nevertheless, the original, relatively high roughness of the XX surfaces cannot be completely eliminated and results in the presence of relatively strong profile variations being superimposed to the previously mentioned pillars.

In terms of comparison, the AB surfaces were also subjected to conventional finishing methods, typically adopted for biomedical devices, i.e., sandblasting carried out with zirconia and corundum. In this respect, it is worth mentioning that LT is based on thermal energy while sandblasting relies on mechanical energy.

In detail, corundum‐blasted surfaces present a smooth appearance: the corundum medium probably plastically deformed the adhering particles, either flattening or removing them. On the contrary, zirconia sandblasting produces a corrugated and shinier surface: XZ and XY conditions were very similar and presented a distribution of non‐uniform ripples.

### 3.2 Morphological Study at 24 H

The initial stages of cell‐biomaterial interaction exert significant influence on subsequent phases of proliferation and differentiation. Cell shape is influenced by the characteristics of the implant surface (topography, chemistry, wettability, hydrophilicity, etc) and quantification of cellular shape can represent a powerful tool to investigate the complex interplay between surfaces and cellular behavior.^[^ ^35^, ^36^ ^]^ The influences of material surface parameters on the biological response remain a complex and contentious subject, with no consensus existing on which of these parameters can consistently and conclusively forecast osteoblast cell behavior, adopting the standard surface finishing techniques.^[^ ^37^ ^]^ Among the several parameters, topography seems to be playing an important role in the earliest protein adsorption event onto materials, which influences cellular interaction and subsequently affects the orientation, adhesion, migration, proliferation, growth, and differentiation of cells.^[^ ^38^ ^]^

**Figure 6:**
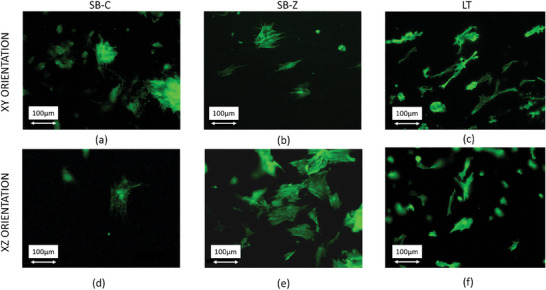
Representative fluorescent images of osteoblast stained with phalloidin following adhesion onto the different surfaces after 24 h. Images were acquired at 20X magnification with an inverted microscope equipped with an epifluorescence setup (Eclipse TiU, NIKON Europe BV, NITAL SpA, Milano, Italy) (Scale bar: 100 µm).

In the current study set‐up, the topographical characteristics (roughness and surface texture) represented the only distinguishing elements among the experimental surfaces during the first 24 h post‐seeding, as the cells were maintained in a growth medium devoid of any differentiation‐inducing agents. Therefore, an analysis of morphology was conducted with fluorescent microscopy green labeling cell cytoskeleton with f‐actin at 24 h after seeding (**Figure** **6**). Specific morphological descriptors were also measured to understand how cells perceive the surface of the samples in the absence of other conditioning factors. The fluorescence images qualitatively highlight a distinct morphology adopted by cells on sandblasted surfaces compared to those seeded on laser‐textured surfaces. Specifically, cells on SB surfaces appear larger and more flattened, while on LT surfaces, cellular elongation prevails, with the presence of protrusions more prominently evident compared to those on SB surfaces.

The quantitative results obtained adopting the morphological descriptors indicate that the micro‐texture achieved through laser texturing influences cell morphology, confirming the qualitative observations. The cell area on LT samples was found to be lower compared to sandblasted samples, regardless of the orientation (**Tables** **4** and **5**). This result aligns with findings by Schnell G et al., who observed similar trends with osteoblast‐like cells MG63 seeded onto nano‐ and microstructured titanium surfaces, as the size and shape of the LT pillars seem to impose limitations on the cell's complete spreading, in contrast to smoother or more regular surfaces.^[^ ^39^ ^]^ Also, Dumas et al., in their investigation of pluripotent mesenchymal stromal cells, observed smaller, branched, and more elongated cells on femtosecond laser‐textured titanium surfaces compared to larger and more spread cells on polished surfaces. These observations were confirmed by the measurement of morphometric parameters, which aligned with our findings: smaller cell area for textured surfaces with a more branched cell shape, confirming a different response of the cellular cytoskeleton to topographic characteristics of the surfaces.^[^ ^40^ ^]^ Despite this smaller cellular area, the morphological descriptors such as roundness, aspect ratio, and circularity indicated an active response of osteoblasts on LT surfaces. Lower values of roundness suggested cellular elongation while a higher aspect ratio was employed in addition to elongation attitude, greater contact with the surface. Values close to 1 for circularity indicated the absence of cellular protrusions (lamellipodia, filopodia, or blebs) along cell boundaries, whereas a decrease in this value toward 0, suggested the formation and/or increase in the number of protrusions. Moreover, the presence of these irregular elements, which enhance cell anchorage phenomena, affected the perimeter, which indeed was comparable between LT and sandblasted surfaces for both orientations. Even Klos found a reduced cell spreading after 24 h from seeding onto laser‐nanostructured surfaces and higher aspect ratios, as in our study, although our values were not statistically different from those obtained for SB surfaces, further confirming the cytoskeletal stimulation by laser surfaces and a tendency to favor differentiation toward the osteoblastic lineage.^[^ ^41^ ^]^

**Table 4:** Morphometric descriptors of osteoblasts shape in response to XY surfaces sandblasted with corundum (SB‐C), zirconia (SB‐Z), and laser textured (LT).

XY	Area [µm^2^]	Perimeter [µm]	Roundness	Aspect Ratio	Circularity
SB‐C	2752 ± [2238,3385]^***^	363 ± [304435]	0.30 ± [0.24,0.37]^*^	2.59 ± [2.09,3.20]	0.32 ± [0.26,0.40]^*^
SB‐Z	2628 ± [2322,2973]^***^	319 ± [290352]	0.33 ± [0.30,0.39]^***^	2.40 ± [2.06,2.80]	0.40 ± [0.35,0.45]^***^
LT	1507 ± [1365,1663]	350 ± [309397]	0.19 ± [0.15,0.24]	3.08 ± [2.58,3.68]	0.21 ± [0.18,0.25]

The measures were performed on at least 30 individual cells manually outlined after f‐actin staining 24 h after seeding. Data are shown as mean and [95% CI]. (SB‐C and SB‐Z vs LT: ^*^, *p* < 0.05; ^**^, *p* < 0.005; ^***^, *p* < 0.001).

**Table 5:** Morphometric descriptors of osteoblast shape in response to XZ surfaces sandblasted with corundum (SB‐C), zirconia (SB‐Z), and laser textured (LT).

XZ	Area [µm^2^]	Perimeter [µm]	Roundness	Aspect Ratio	Circularity
SB‐C	3549 ± [3052, 4128]^***^	406 ± [327, 504]	0.23 ± [0.19,0.27]^#^	2.41 ± [2.18,2.66]	0.26 ± [0.21,0.31]
SB‐Z	2650 ± [2256,3112]^***^	302 ± [269, 338]	0.31 ± [0.27,0.36]^***^	2.46 ±[2.10,2.87]	0.36 ±[0.32,0.40]^***^
LT	1382 ± [1130,1690]	280 ± [234, 310]	0.19 ± [0.16,0.23]	3.30 ± [2.53,4.30]	0.24 ± [0.20,0.29]

The measures were performed on at least 30 individual cells manually outlined after f‐actin staining 24 h after seeding. Data are shown as mean and [95% CI]. (SB‐C and SB‐Z vs LT: ^*^, *p* < 0.05; ^**^, *p* < 0.005; ^***^, *p* < 0.001; SB‐C vs SB‐Z: *^#^*, *p* < 0.05).

The orientation of titanium deposition (XY and XZ) did not statistically significantly influence the analyzed morphological descriptors, as demonstrated by the graphs presented in Panel S1 (Supporting Information). The quantitative morphometry results indicated a surface treatment‐dependent modulation of osteoblast morphogenesis, revealing a significant impact exerted by the LT surface texturing on osteoblast morphological behavior in the early stages of adhesion.

### 3.3 Cell Metabolic Activity

**Figure 7:**
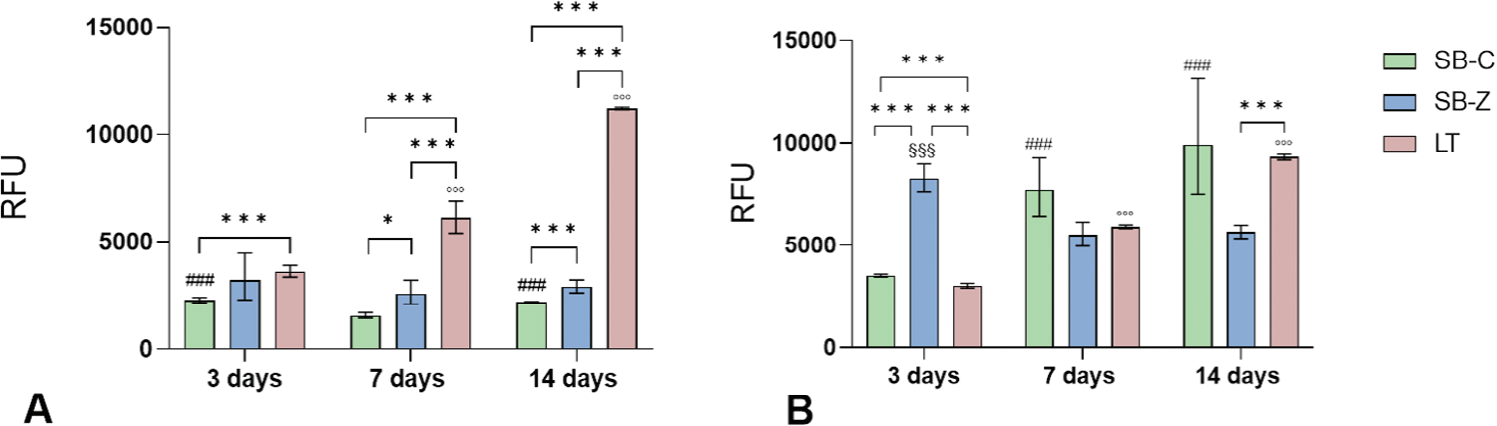
Metabolic activity was measured with Alamar Blue assay at 3, 7, and 14 days of culture of osteoblasts seeded onto different surfaces finishing conditions realized onto titanium samples with a) XY orientation or b) XZ orientation. The results are given as relative fluorescent units (RFU) and values are reported as mean and [95% CI] obtained from four replicate materials. Statistical comparison is reported in the graphs: among experimental times within the same material three different symbols were used: ^#^ for SB‐C; ^§^ for SB‐Z and ^°^ for LT (for example ^#^, *p* < 0.05; ^##^, *p* < 0.005; ^###^, *p* < 0.001) and among experimental materials within the same experimental time (^*^, *p* < 0.05; ^**^, *p* < 0.005; ^***^, *p* < 0.001).

In terms of metabolic activity, as measured by Alamar Blue assay, all investigated surfaces demonstrated their ability to support osteoblasts metabolism, albeit with some notable differences. In the XY orientation, sandblasting with both types of particles did not induce a significant increase in metabolic activity during the selected experimental times exhibiting constant RFU values. Only slight but significant differences were observed for SB‐C between 3 days versus 7 days (*p* < 0.001) and between 14 days versus 7 days (*p* < 0.001). No differences were detected for SB‐Z samples over time. On the contrary, the LT finishing technique seemed to better support cell metabolic activity on XY orientation than sandblasted counterparts over time (LT‐7 days vs LT‐3 days, *p* < 0.001; LT‐14 days vs LT‐3, and vs LT‐7 days *p* < 0.001) and in significance versus sandblasted surfaces within the same experimental time as showed in **Figure** **7**a. The profilometry analysis reveals that the surface roughness of the AB materials obtained with XY printing orientation is consistently regular and relatively smooth. The subsequent surface SB procedure appears to be insufficient in significantly enhancing the roughness of the XY surface, thereby failing to stimulate cell metabolic activity to a significant extent. Conversely, laser treatment introduces microroughness which markedly stimulates cells. As previously stated, there is no global consensus on which surface roughness or texture parameters effectively guide cellular behavior.^[^ ^37^ ^]^ Literature often presents conflicting data, although there are in vitro indications that roughness, within certain micro and nanometers, can influence positively the behavior of cells favoring their commitment, differentiation, and maturation process into osteoblasts.^[^ ^42^, ^43^ ^]^

In our study, the effect exerted by roughness is further confirmed by the trend of data on XZ surfaces (Figure 7b). The SB finishing creates a surface roughness able to increase metabolic activity for SB‐C in a significant manner between 3 and 7 days (*p *< 0.001). For the SB‐Z, the observed trend was less regular compared to SB‐C, with significant metabolic activity measured at 3 days compared to that obtained at 7 days and 14 days (*p *< 0.001). Regarding LT‐ surfaces, the values of the metabolic activity increase over time in a comparable way to that observed for SB‐C, with statistical significances among times: 7 days versus 3 days, and 14 days versus 3 days and versus 7 days at the same level of significance (*p *< 0.001).

For the XZ orientation, the trend is probably reversed from those observed for XY. On a smooth surface like the as‐built XY, the use of the laser texturing introduces a favorable roughness for osteoblast activity, whereas on a surface such as XZ, already endowed with inherent roughness as showed by AB profilometry (Figures 4 and 5), the laser may not lead to a significant advantage in term of metabolic activity enhancement, as in some areas, the resulting roughness values are perhaps too high to be perceived positively by osteoblast. These results are further confirmed by the statistical analysis performed comparing the two orientations (Panel S2, Supporting Information): XZ orientation associated with both SB finishing stimulates cells metabolic activity with a greater extent in comparison to XY, while for LT surfaces, the most performing orientation is XY thus confirming the influence exerted by topography of the starting surfaces (as built).

### 3.4 Scanning Electron Microscopy Results

**Figure 8:**
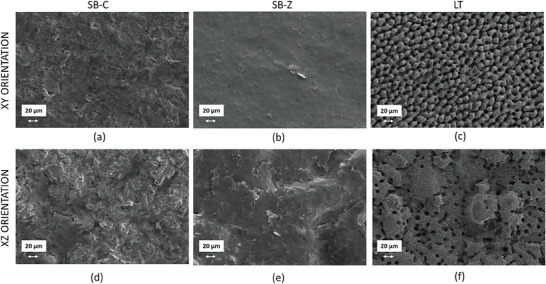
Representative photomicrographs acquired using scanning electron microscopy showing the osteoblast spreading at 7 days onto the two orientations surface finished with two techniques represented by sandblasting with corundum (SB‐C)(a)(d) or zirconia (SB‐Z) (b)(e) and laser texturing (LT)(c)(f).

To evaluate the morphology of osteoblast cells in the context of the various surface finishes studied, SEM was performed at both 7 and 14 days of cultusre (**Figure** **8**). On sandblasted surfaces, osteoblastic cells exhibited a flattened morphology, evenly spreading across the surface while firmly adhering to the underlying substrate. On the XY surface, only the presence of cytoplasmic extensions connecting with other cells and the substrate allows for clearer identification (**Figure** **8**a,b), while the pronounced roughness of the XZ surfaces enables sharper visual distinction (Figure 8d,e). The thinness of the cells, particularly evident in corundum blasting, revealed the underlying titanium surface in both orientations. The appearance of laser surfaces differed significantly (Figure 8c,f). Due to laser micro‐ and nano‐texturing, cells cultured on both orientations were not as thin as those observed on sandblasted surfaces. Instead, they appeared with a solid 3D appearance and were situated within cavities or spread along the elevations of the structures created by laser ablation. In these instances, the cells visibly conformed to the surface structure, lining the pillars or craters with more distinct protoplasmic processes along their boundaries. This behavior has been previously observed by other authors facing laser‐textured surfaces, as also discussed in the morphological descriptors section.^[^ ^39^ ^]^ These authors observed with SEM that cells adhering to rough surfaces created by laser texturing exhibited less tightly adhesion and appeared more dispersed across the surface especially when the surface roughness was very high with Sa values of 2.69 ± 0.10 µm.

### 3.5 Gene Expression and Immunoenzymatic Assays

To explore how surface finishing affects osteoblast activity, particularly in terms of differentiation capabilities, we examined the gene expression and protein synthesis of key specific markers associated with osteogenic differentiation commitment. The analysis initially centered on the gene expression of two pivotal growth factors: Transforming growth factor β1 (*TGF‐β1*) and Bone Morphogenetic Protein 2 (*BMP2*). Both are essential in the bone repair and regeneration process, as they stimulate the proliferation and differentiation of osteoprogenitor cells.^[^ ^44^ ^]^

**Figure 9:**
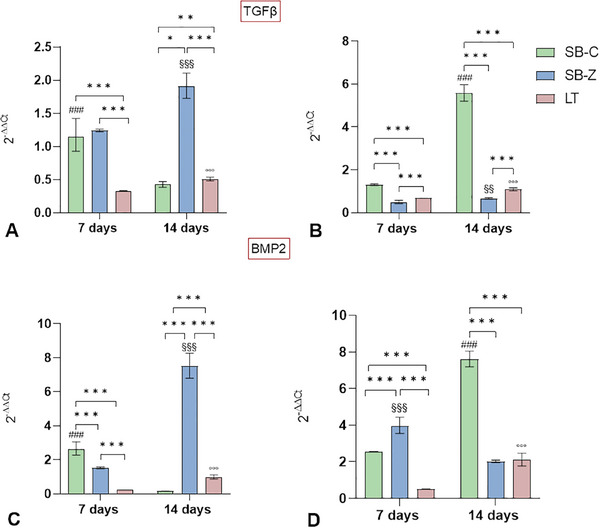
TGF‐β1 and BMP‐2 gene expression at 7 and 14 days of culture of osteoblasts seeded onto different surfaces finishing conditions realized onto titanium samples with XY orientation (a and c) or XZ orientation (b and d). The results show the mean and 95% CI obtained from three replicate materials. Statistical analysis is reported in the graphs: between experimental times in the same material, three different symbols were used: # for SB‐C; § for SB‐Z, and ° for LT (for example ^###^, *p* < 0.001; ^##^, *p* < 0.005; ^#^, *p* < 0.05) and among experimental materials in the same experimental time (^***^, *p* < 0.001; ^**^, *p* < 0.005; ^*^, *p* < 0.05).

For all the surfaces analyzed, we observed expression of the TGF‐β1 gene. Specifically, in the XY orientation (**Figure** **9**a), the sandblasting approach appears to promote the upregulation of this gene. SB‐Z leads to a significant upregulation of *TGF‐β1*, with expression increasing from 7 to 14 days (*p* < 0.001), while for SB‐C an earlier up‐regulation was detected at 7 days, which significantly decreased by 14 days (*p* < 0.001). On femtosecond laser‐textured surfaces, the expression of *TGF‐β1* shows significant peaks at 14 days compared to 7 days (*p* < 0.001), even though the value obtained at 7 days was lower compared to those obtained for both sandblasted materials (*p* < 0.001). Additionally, at 14 days, SB‐Z was significantly higher than the LT surface (*p* < 0.001).

In the XZ orientation (Figure 9b), sandblasting with corundum significantly upregulates the gene, with a notable peak at 14 days (*p* < 0.001). At the same level of significance, surfaces sandblasted with zirconia and LT induced an up‐regulation of the gene, although the fold increase was not as consistent as that observed for corundum. At both 7 and 14 days, the XY orientation influenced the expression of *TGF‐β1* by SB‐Z, while the XZ orientation favored LT. Orientation did not influence SB‐C at 7 days, but it did significantly influence it at 14 days with XZ orientation (Panel S3, Supporting Information).

Differences in the expression of BMP2 have also been detected, exhibiting a similar trend of expression as observed for *TGF‐β1*. This consistent trend was observed across all surfaces in the XY orientation (Figure 9c), confirming the strong interrelation that exists between these two growth factors. In the XZ orientation, SB‐C still emerges as the surface finishing capable of stimulating increased expression over time (SB‐C: 14 days vs 7 days, *p *< 0.001). However, SB‐Z promotes an earlier *BMP2* expression (SB‐Z:7 days vs 14 days, *p* < 0.001), compared to SB‐C and LT surfaces (*p* < 0.001). Therefore, SB‐Z induces BMP2 expression with less effectiveness compared to SB‐C, but at an earlier stage. Regarding *BMP2*, XZ orientation primarily influences the gene expression, except at 14 days for SB‐z, where the XY orientation favors gene up‐regulation.

The expression of these two pivotal genes on the LT surfaces of both orientations did not result in significant up‐regulations at the investigated experimental times, compared to that observed for sandblasted surfaces. This trend may be attributed to an earlier up‐regulation for cells seeded on the LT surfaces, possibly related to their unique microtexture. However, comparing with existing literature to verify our hypothesis is challenging due to the limited evidence available on the biological properties of LT surfaces. As observed with morphological descriptors, gene expression also reveals a different behavior between SB and LT. To delve deeper into this aspect, specific osteogenic markers associated with differentiation and maturation, including ALPL, *COL1A1*, and *SPP1*, were analyzed following the examination of *TGF‐β1* and *BMP2*. Although a peak in up‐regulation may have already subsided, the activation of other key downstream genes in the *BMP2* and *TGF‐β1* cascade could still be effective and observable, potentially explaining the values observed for LT surfaces.

Particularly, *BMP2* is a growth factor strongly associated with osteoblastogenesis through the activation of the Smad signaling pathway. This pathway can lead to the expression of alkaline phosphatase and osteocalcin. Additionally, *BMP2* induces the expression of Osterix (SP7), which in turn triggers homeobox 5 (DLX5), potentially resulting in the induction of osteopontin expression and further enhancing alkaline phosphatase expression.^[^ ^45^ ^]^

Alkaline Phosphatase (*ALPL*), collagen type‐1 (*COL1A1*), and osteopontin (*SPP1*) genes serve as typical markers of osteoblast differentiation and maturation. *ALPL* gene holds particular significance as a marker indicating the mineralization phase of bone matrix, suggesting an early osteoblast differentiation and participation in establishing the bone extracellular matrix for subsequent mineral tissue deposition.^[^ ^46^ ^]^ The *SPP1* gene encodes one of the most prevalent non‐collagenous proteins in the extracellular matrix produced by osteoblasts and its expression is linked to a later stage of differentiation.^[^ ^47^ ^]^ It plays a role in bone mineralization and facilitates cell adhesion to surfaces, while the *COL1A1* gene encodes the major component of type I collagen, the primary protein of the bone matrix its expression is consistent with the transition from an early to a more advanced stage of cellular maturation.^[^ ^48^ ^]^ The presence and expression of these specific genes by cells first indicate that both sandblasted and laser‐textured surfaces are capable of sustaining the maintenance of osteoblast phenotypic characteristics.

**Figure 10:**
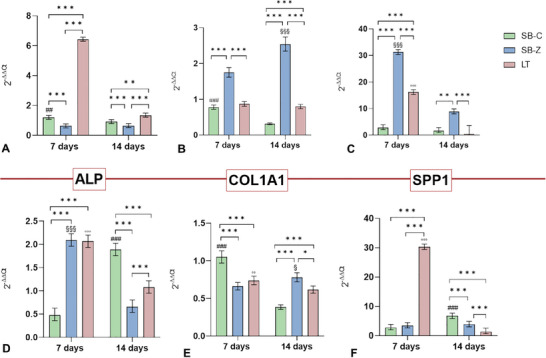
*ALPL, COL1A1*, and *SSP1* gene expression at 7 and 14 days of culture of osteoblasts seeded onto different surfaces finishing conditions realized onto titanium samples with XY orientation (a‐c) or XZ orientation (d‐f). The results show the mean and 95% CI obtained from three replicate materials. Statistical analysis is reported in the graphs: between experimental times in the same material three different symbols were used: # for SB‐C; § for SB‐Z and ° for LT (for example: ^###^, *p* < 0.001; ^##^, *p* < 0.005; ^#^, *p* < 0.05) and among experimental materials in the same experimental time (^***^: *p* < 0.001; ^**^: *p* < 0.005; ^*^: *p* < 0.05).

As hypothesized, in the XY orientation, LT surfaces induced a significant up‐regulation of the *ALPL* gene at 7 days, which decreased by 14 days (LT 7 days vs 14 days, *p* < 0.001). In contrast, the up‐regulation of this gene on both sandblasted surfaces was not as pronounced (**Figure** **10**a). In the XZ orientation (Figure 10d), both SB‐Z and LT surfaces induced a similar up‐regulation at 7 days (LT: 7 days vs 14 days, *p*<0.001; SB‐Z: 7 days vs 14 days, *p*<0.001), while the peak for SB‐C was detected at 14 days (SB‐C: 14 days vs 7 days, *p*<0.001).

Regarding *COL1A1* results, for SB surfaces with XY orientation (Figure 10b), zirconia sandblasting promoted a significant up‐regulation especially at 14 days compared to the other conditions (SB‐Z vs SB‐C and LT at 7 days and 14 days, *p*<0.001). Comparable values were detected for SB‐C and LT at 7 days and a significance for LT in comparison to SB‐C (*p*<0.001) was present at 14 days. Corundum sandblasting was most efficient in stimulating a *COL1A1* gene response on surfaces with XZ orientation at 7 days (SB‐C vs SB‐Z and LT, *p*<0.001), while at 14 days the best performance was attributable to zirconia (Figure 10e).

Finally, the expression of *SPP1* was investigated, again finding that SB‐Z in XY orientation best stimulated the up‐regulation of this gene at both experimental times (SB‐Z vs SB‐C and LT at 7 days and 14 days, *p*<0.001) (Figure 10c). Laser texturing also induced a significant up‐regulation at 7 days, although less pronounced. However, for the XZ orientation, only the LT surfaces led to a significant upregulation of SPP1 at 7 days (LT vs SB‐C and SB, *p*<0.001), followed by a decrease at 14 days. Sandblasted surfaces did not show a noticeable increase in this gene expression (Figure 10f).

The orientation of titanium powder deposition significantly influences this gene expression, reflecting the peak of up‐regulation of different genes. For example, for ALPL, the XY orientation significantly influences gene expression for LT surfaces at 7 days. Similarly, the XZ orientation has a significant influence on ALPL expression for SB‐Z at 7 days (Panel S4, Supporting Information).

Different surface morphologies were directly perceived by the cells and were also able to influence the synthesis and release of key proteins involved in promoting, accelerating, or interfering with the process of osseointegration. Therefore, the commitment of osteoblasts induced by surface topography was also verified by performing an enzyme‐linked immunosorbent assay (ELISA) to quantitatively detect specific early and late analytes within the culture supernatant.

The first investigated analyte was represented by collagen type I, the most abundant protein in the extracellular matrix, which serves as a scaffold for bone mineral deposition. Collagen expression and synthesis start early and continue during the mineralization phase, representing a crucial event in the biological response cascade. For both XZ and XY orientations, collagen production, and supernatant release were detected in all experimental conditions to varying extents. On XY orientation (**Table** **6**), the LT surface induced a constant and incremental production and release during the experimental times, while cells seeded onto sandblasted materials exhibited a trend of synthesis that was less regular and constant compared to what was obtained by LT. Similarly, on XZ orientation (**Table** **7**), the LT surfaces exhibit the most regular trend over time, increasing during experimental time points, followed by SB‐C. Onto SB‐Z the highest peak of synthesis was observed at 3 days followed by a gradual decrease at 7 days and 14 days.

**Table 6:** Quantitative results of ELISA assays performed onto osteoblasts supernatant in response to XY surfaces sandblasted with corundum (SB‐C), zirconia (SB‐Z), and laser textured (LT).

	3 days	7 days	14 days
Collagen‐type I [ng mL^−1^]			
SB‐C	1.78 ± [1.65−1.92]	9.22 ± [8.85−9.60] ^###, ***^	5.30 ± [4.52−6.21]^###^
SB‐Z	11.30 ± [10.57−12.07] ^§§§, ***^	10.86 ± [10.31−11.44] ^§§§, ***^	6.24 ± [5.76−6.76]
LT	4.55 ± [3.67−5.65] ^***^	5.73 ± [5.47−6.00]	12.00 ± [11.27−14.99] ^°°°, ***^

Osteonectin‐ SPARC [ng mL^−1^]			
SB‐C	1.92 ± [1.70−2.16]	13.94 ± [11.49−16.96]^###,***^	2.62 ± [2.50−2.76] ^###, ***^
SB‐Z	10.89 ± [10.62−11.16]^§§§^, ^***^	31.80 ± [27.67−36.53] ^§§§, ***^	6.05 ± [4.93−7.43]^***^
LT	1.75±[1.74−1.75]^°°°^	2.12 ± [2.10−2.14]^°°°^	1.59 ± [1.60−1.61]

Osteocalcin [ng mL^−1^]			
SB‐C	/	9.01 ± [7.71−10.53] ^***^	8.18 ± [6.75−9.91] ^**^
SB‐Z	/	1.83 ± [1.78−1.89]	4.87± [4.57− 5.18] ^§§§^
LT	/	10.81 ± [10.26−11.40] ^***^	13.18 ± [12.79−13.61] ^°°°, ***^

Interleukin‐1β [pg mL^−1^]			
SB‐C	11.67 ± [7.14, 19.06]	15.76 ± [10.96, 22.67] ^*^	2.11 ± [0.14, 31.79]
SB‐Z	45.25 ± [39.87, 51.36] ^§§§,***^	20.41 ± [15.41, 27.02] ^**^	20.95 ± [15.94, 27.53] ^**^
LT	2.57 ± [0.28, 23.86]	2.33 ± [0.20, 27.16]	2.61 ± [0.29, 23.28] ^°^

Interleukin‐6 [pg mL^−1^]			
SB‐C	24.79 ± [21.58, 28.47] ^###,***^	15.04 ±[13.10, 17.28] ^***^	14.60 ± [12.70, 16.76] ^***^
SB‐Z	18.93 ± [16.48, 21.74] ^***^	15.35 ± [13.37, 17.63]^***^	13.91 ± [12.11, 15.97]^***^
LT	1.33 ± [1.16, 1.53] ^°°°^	0.65 ± [0.56, 0.74]	8.85 ± [7.71, 10.16] ^°°°^

Data are shown as mean and 95% CI. Among experimental times in the same material, three different symbols were used: # for SB‐C; § for SB‐Z, and ° for LT (for example ^###^, *p* < 0.001; ^##^, *p* < 0.005; ^#^, *p* < 0.05) and among experimental materials in the same experimental time (^***^, *p* < 0.001; ^**^, *p* < 0.005; ^*^, *p* < 0.05).

**Table 7:** Quantitative results of ELISA assays performed onto osteoblast's supernatant in response to XZ surfaces sandblasted with corundum (SB‐C), zirconia (SB‐Z), and laser textured (LT).

	3 days	7 days	14 days
Collagen‐type I [ng mL^−1^]			
SB‐C	5.00 ± [3.20−7.80]	11.02 ± [9.49−12.81] ^##^	8.96 ± [8.28−9.70] ^#,***^
SB‐Z	10.26 ± [9.64−10.91] ^§, §§§, **,***^	9.05 ± [8.82−9.27] ^§§§^	2.87 ± [1.43−5.75]
LT	6.37 ± [5.30−7.68]	9.16 ± [8.86−9.46]^°°°^	12.99 ± [11.27−14.99]^°°,°°°,***, **^

Osteonectin –SPARC [ng mL^−1^]			
SB‐C	1.82 ± [1.71−1.94]	32.86 ± [29.86−36.15] ^###,***^	24.53 ± [21.39−28.12] ^###, ***^
SB‐Z	10.09 ± [9.91−10.27]^§§§, ***^	6.46 ± [3.24−12.90]	22.20 ± [16.55−29.76] ^§§§,§§,***^
LT	1.85 ± [1.84−1.85] ^°°°^	1.60 ± [1.57−1.60]	1.62 ± [1.60−1.64]

Osteocalcin [ng mL^−1^]			
SB‐C	/	8.68 ± [7.64−9.86] ^***^	8.20 ± [7.40−9.10]
SB‐Z	/	1.74 ± [1.72−1.77]	7.84 ± [6.85−8.98] ^§§§^
LT	/	8.65 ± [8.19−9.12] ^***^	15.30 ± [14.67−15.97] ^°°°, ***^

Interleukin‐1β [pg mL^−1^]			
SB‐C	11.67 ± [7.14, 16.06]	17.68 ± [10.92, 22.67]	12.22 ± [8.14, 31.80] ^**^
SB‐Z	39.52 ± [29.87, 51.36]^§§§, ***^	13.85 ± [10.41, 27.01] ^**^	1.84 ± [0.94, 2.88]
LT	2.46 ± [0.27, 28.86]	2.22 ± [0.20, 27.16]	2.48 ± [0.29, 23.33]

Interleukin‐6 [pg mL^−1^]			
SB‐C	22.54 ± [19.20, 25.42] ^#, ***^	17.41 ± [15.44, 19.63] ^***^	16.04 ± [14.22, 18.08]^***^
SB‐Z	24.26 ± [21.51, 27.35] ^§§§,***^	15.13 ± [13.42, 17.05] ^***^	14.55 ± [12.91, 16.41]^***^
LT	1.36 ± [1.21, 1.53]^°°°^	0.83± [0.73, 0.93]	5.40 ± [4.75, 6.05] ^°°°^

Data are shown as mean and 95% CI. Among experimental times in the same material, three different symbols were used: # for SB‐C; § for SB‐Z, and ° for LT (for example ^###^, *p* < 0.001; ^##^, *p* < 0.005; ^#^, *p* < 0.05) and among experimental materials in the same experimental time (^***^, *p* < 0.001; ^**^, *p* < 0.005; ^*^, *p* < 0.05).

The second analyte investigated was osteonectin, also known as SPARC (secreted protein acidic and rich in cysteine), which represents one of the most important non‐collagenous proteins. It is used as a late marker of osteoblastic functional differentiation as it is involved in the differentiation, maturation, and mineralization steps of osteoblast.^[^ ^49^ ^]^ The trend observed for surfaces in the XY orientation shows that SB‐Z leads to a significant osteonectin production at 7 days, followed by corundum, while at 14 days, both sandblasted surfaces experience a sharp decline in protein production. LT surfaces on XY orientation, however, show a minimal synthesis of this protein compared to SB surfaces.

Regarding the XZ orientation, sandblasted surfaces exhibit markedly lower production and release compared to LT surfaces, following a trend comparable to that observed for the XY orientation. Lt surfaces show the lowest amount of osteonectin protein. Given the low synthesis of osteonectin, especially for LT surfaces in both orientations, another late marker of differentiation was investigated focusing on 7 and 14 days of synthesis, to better understand the differentiation phase of osteoblasts.

Osteocalcin, another key protein present throughout the step of bone tissue formation, especially in the processes of matrix mineralization, was therefore quantified to verify the maintenance of the osteoblastic phenotype. Indeed, it is frequently investigated as a late marker in the differentiation pathway, and it tends to increase when cells begin to reduce their expression and synthesis of osteonectin, suggesting the progression of the cellular differentiation process while maintaining the osteoblastic phenotype.

The results obtained confirmed our hypothesis, showing high expression of this protein with an increasing trend at the two experimental time points for LT surfaces in both deposition orientations. This suggests the maintenance of the osteoblastic phenotype stimulated by this peculiar nano/microstructured textured surface and also the significant stimulation of the mineralization process. Generally, the results regarding protein synthesis indicate that all investigated surfaces were able to stimulate the production and release of key proteins with differences clearly linked to the distinct surface topography and roughness (Panel S5, Supporting Information).

Apart from anabolic cytokines, also catabolic cytokines can provide information as they represent important signaling molecules produced by the cells, mainly focused on the activation of pathways that break down the bone matrix components. Interleukin 1β production was minimal onto each surface of both orientations except for the value found for the SB‐Z surfaces at the first experimental time point of 3 days, which then gradually decreased in the two subsequent experimental time points probably attributable to the presence onto surfaces of zirconia residues. A similar trend was also observed for IL6, with a peak of production observed at the first experimental time for both orientations onto both sandblasting surfaces, followed by a significant decrease at 7 and 14 days. As for IL‐1β, the production and release of IL‐6 were significantly lower on LT surfaces compared to sandblasting surfaces, suggesting other potential advantages of this type of surface finishing approach, which avoids the use of powder and polishing techniques to obtain suitable roughness for bone tissue connection. However, for IL‐6, an increase is observed at 14 days for LT surfaces with both orientations, still lower than the sandblasted surfaces, which is compatible with the high levels of proliferation and cell growth concentrated on these surfaces, widely colonized as shown by SEM images and with a string metabolic activity as showed by Alamar Blue (Panel S6, Supporting Information).

**Collagen Type‐1**: Among experimental times in the same material: SB‐C: ^###^ 7 days versus 3 and 14 days; ^###^ 14 days versus 3 days; SB‐Z: ^§§§^ 3 and 7 days versus 14 days; LT: ^°°°^ 14 days versus 3 and 7 days; Among experimental materials in the same experimental time: 3 days: ^***^ SB‐C versus SB‐Z and LT; ^***^ LT versus SB‐Z; 7 days: ^***^ SB‐Z versus SB‐C and LT; ^***^ SB‐C versus LT; 14 days: ^***^ LT versus SB‐Z and SB‐C.

**Osteonectin**: Among experimental times in the same material: SB‐C: ^###^ 7 days versus 3 and 14 days; ^###^ 14 days versus 3 days SB‐Z: ^§§§^ 7 days versus 3 days; ^§§§^ 3 days versus 14 days; LT: ^°°°^ 7 days versus 3 and 14 days; ^°°°^ 3 days versus 14 days. Among experimental materials in the same experimental time: 3 days: ^***^ SB‐Z versus SB‐C and LT; 7 days: ^***^ SB‐Z versus SB‐C and LT; ^***^ SB‐C versus LT; 14 days: ^***^ SB‐Z versus SB‐C and LT; ^***^ SB‐C versus LT.

**Osteocalcin**: Between experimental times in the same material: SB‐C; SB‐Z: ^§§§^ 14 days versus 7 days; LT: ^°°°^14 days versus 7 days. Among experimental materials in the same experimental time: 7 days: ^***^ SB‐C versus SB‐Z; 7 days; ^***^ LT versus SB‐Z; 14 days: ^***^ LT versus SB‐C and SB‐Z; ^**^ SB‐C versus SB‐Z.

**IL‐1β**: Among experimental times in the same material: LT: ^°^ 14 days versus 7 days; SB‐Z: ^§§§^ 3 days versus 7 and 14 days. Among experimental materials in the same experimental time: 3 days: ^***^ SB‐Z versus SB‐C and LT; 7 days: ^*^ SB‐C versus LT; ^**^ SB‐Z versus LT; 14 days: ^**^ SB‐Z versus SB‐C and LT.

**IL‐6**: Among experimental times in the same material: SB‐C: ^###^ 3 days versus 7 and 14 days; LT: ^°°°^ 14 days versus 3 and 7 days; ^°°°^ 3 days versus 7 days. Among experimental materials in the same experimental time: 3, 7, and 14 days: ^***^ SB‐C and SB‐Z versus LT

**Collagen Type‐1**: Among experimental times in the same material: SB‐C: ^##^ 7 days versus 3 days; ^#^ 14 days versus 3 days; SB‐Z: ^§^ 3 days versus 7 days; ^§§§^ 3 and 7 days versus 14 days LT: ^°°°^ 14 and 7 days versus 3 days; ^°°^ 14 days versus 7 days Among experimental materials in the same experimental time: 3 days: ^**^ SB‐Z versus SB‐C; ^***^ SB‐Z versus LT; 14 days: ^***^ LT versus SB‐Z; ^**^ LT versus SB‐C and ^***^ SB‐C versus SB‐Z.

**Osteonectin**: Among experimental times in the same material: SB‐C: ^###^ 7 days versus 3 and 14 days; ^###^ 14 days versus 3 days SB‐Z: ^§§§^ 14 days versus 3 days; ^§§^ 14 days versus 7 days; ^§§§^ 3 days versus 7 days; LT: ^°°°^ 3 days versus 7 and 14 days. Among experimental materials in the same experimental time: 3 days: ^***^ SB‐Z versus SB‐C and LT; 7 days: ^***^ SB‐C versus SB‐Z and LT; 14 days: ^***^ SB‐C and SB‐Z versus LT.

**Osteocalcin**: Among experimental times in the same material: SB‐Z: ^§§§^ 14 days versus 7 days; ^°°°^ 14 days versus 7 days. Among experimental materials in the same experimental time: 7 days: ^***^ SB‐C versus SB‐Z; 7 days; ^***^ LT versus SB‐Z; 14 days: ^***^ LT versus SB‐C and SB‐Z;

**IL‐1β**: Among experimental times in the same material: SB‐Z: ^§§§^ 3 days versus 7 and 14 days; Among experimental materials in the same experimental time: 3 days: ^***^ SB‐Z versus SB‐C and LT; 7 days: ^**^ SB‐Z versus LT; 14 days: ^**^ SB‐C versus SB‐Z and LT.

**IL‐6**: Among experimental times in the same material: SB‐C: ^#^ 3 days versus 14 days; SB‐Z: ^§§§^ 3 days versus 7 and 14 days; LT: ^°°°^ 14 days versus 3 and 7 days; ^°°°^ 3 days versus 7 days. Among experimental materials in the same experimental time: 3, 7, and 14 days: ^***^ SB‐C and SB‐Z versus LT and at 14 days ^***^ SB‐C versus SB‐Z

The present results confirm how surface topography, in terms of appropriate roughness, can influence osteoblast fate and function, overshadowing the impact of other surface characteristics in the adopted experimental setup and investigated parameters. Additive manufacturing techniques, such as LFBP, when combined with appropriate surface texturing, could significantly improve the possibility of creating biomaterials with instructive properties to direct cellular behavior. In particular, laser texturing shows interesting characteristics in terms of topographies that can be realized, capable of promoting cellular differentiation and maturation. We also observed that the deposition orientation, whether XY or XZ, influences the modulation of various biological activities, such as metabolic activity, and to a lesser extent, gene expression and protein synthesis. Overall, the XZ orientation exerts a greater influence on the various parameters compared to XY, as the as‐built material, with already pronounced topographic characteristics, makes the effects of finishing treatments more evident. This factor does not seem to affect cell shape in the very early stages of adhesion, for which surface finishing and related roughness provide the most instructive cues.

## 4 Conclusion

The advent of LPBF techniques has undoubtedly made a significant impact in the biomedical field, enabling the production of customized additively manufactured materials tailored to match individual patient characteristics and needs.

This work represents one of the first evidence regarding the biological effect exerted by technology combinations. The main results can be summarized as follows:

- Promising potentiality in using deep LT as post‐processing of Ti6Al4V samples fabricated by LPBF process;
- Dimples and peculiar morphologies obtained after LT confirm the possibility of tailoring the surface in order to address the enhanced specific cell growth;
- Good biocompatibility results for all tested surfaces;
- Laser texturing is able to introduce favorable and instructive surface characteristics for osteoblast fate and function, thus representing a very promising and potential tool to tailor surfaces.

All these findings must be confirmed with more complex preclinical studies which deepen the advantages and disadvantages offered by this technique. The evidence derived from the present study has demonstrated the biocompatibility of all tested surfaces and regarding LT its potential in enhancing osteoblast primary cells maturation and differentiation thus providing a new possible approach to improving clinical implant success in the future.

## Conflict of Interest

The authors declare no conflict of interest.

## Supporting information



Supporting Information

## Data Availability

The data that support the findings of this study are available from the corresponding author upon reasonable request.
