# Supplementary material for: Biological Characterization of Ti6Al4V Additively Manufactured Surfaces: Comparison Between Ultrashort Laser Texturing and Conventional Post‐Processing
Source: Adv Healthc Mater. 2024 Oct 22;14(4):2402873. doi: 10.1002/adhm.202402873 (PMC11804838; doi:10.1002/adhm.202402873)

ADVANCED HEALTHCARE MATERIALS

Supporting Information

for *Adv. Healthcare Mater.*, DOI 10.1002/adhm.202402873

Biological Characterization of Ti6Al4V Additively Manufactured Surfaces: Comparison Between Ultrashort Laser Texturing and Conventional Post-Processing

Maria Sartori, Chiara Bregoli, Melania Carniato, Luca Cavazza, Melania Maglio, Gianluca Giavaresi, Carlo Alberto Biffi, Jacopo Fiocchi, Emanuele Gruppioni, Ausonio Tuissi and Milena Fini*

PANEL 1. **Morphometric descriptors of osteoblasts shape in response to XY or XZ of materials.**
The measures were performed onto at least 30 individual cells manually outlined after f-actin staining at 24 hours after seeding. Statistical analysis is reported in the graphs, between materials with the same types of surface finishing and different orientation three different symbols were used: # for SB-C; § for SB-Z and ° for LT (for example $^{\#\#\#}$, $p < 0.001$; $^{\#\#}$, $p < 0.005$; $^{\#}$, $p < 0.05$).

- **(A)** SB-C, SB-Z, LT: for all materials the orientation does not statistically significantly influence cells area.
- **(B)** SB-C, SB-Z, LT for all materials the orientation does not statistically significantly influence cells perimeter.
- **(C)** SB-C, SB-Z, LT: for all materials the orientation does not statistically significantly influence cells roundness.
- **(D)** SB-C, SB-Z, LT: for all materials the orientation does not statistically significantly influence cells aspect ratio.
- **(E)** SB-C, SB-Z, LT: for all materials the orientation does not statistically significantly influence cells circularity

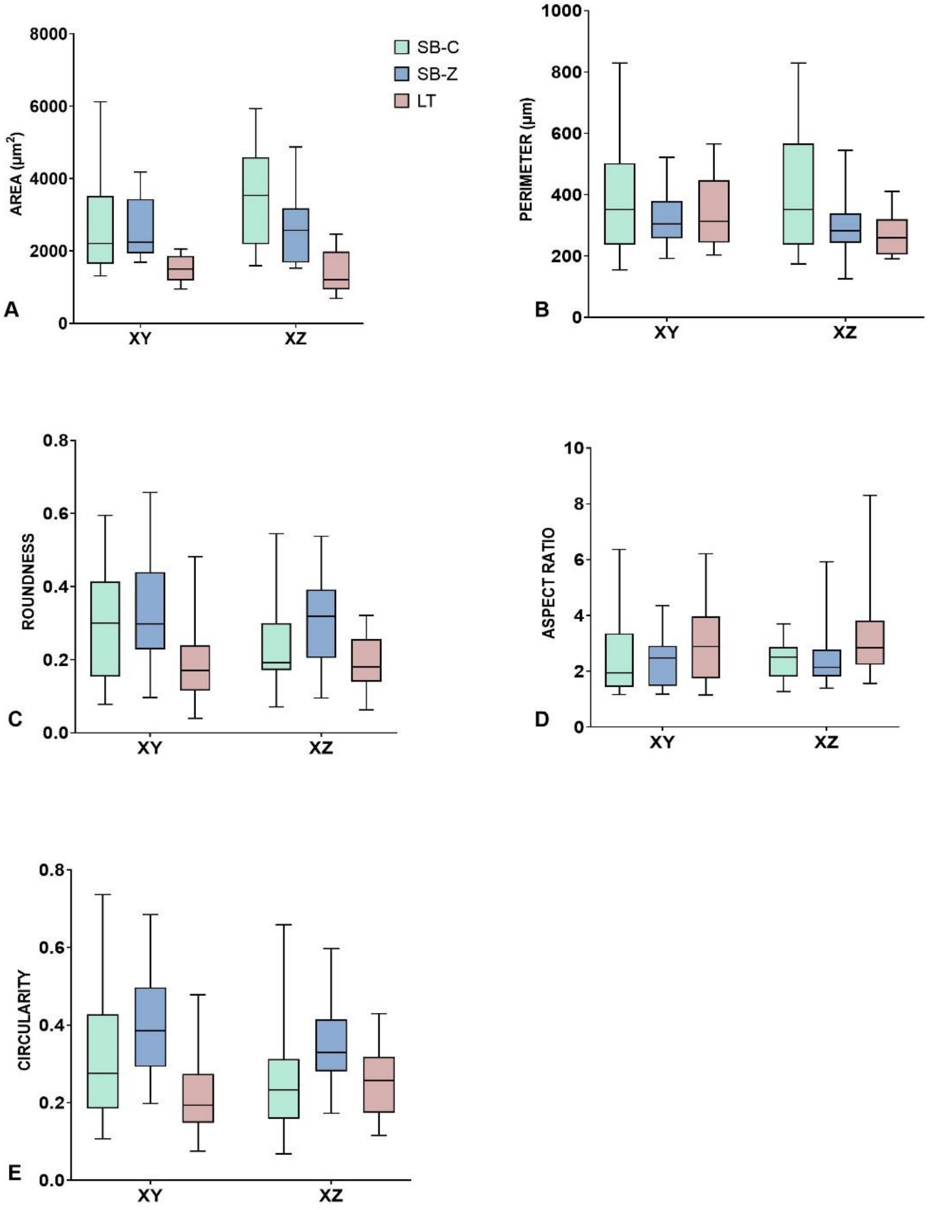

PANEL 2. Metabolic activity measured with **Alamar Blue** assay at 3, 7 and 14 days of culture of osteoblasts seeded onto different surfaces finishing conditions realized onto titanium samples with XY orientation (A) or XZ orientation (B). The results are given as relative fluorescent units (RFU) and values are reported as mean ± and [95% CI] obtained from four replicate materials. Statistical analysis is reported in the graphs: between materials with the same types of surface finishing and different orientation at the same experimental time, three different symbols were used: # for SB-C; § for SB-Z and ° for LT (for example $^{\#\#\#}$, $p < 0.001$; $^{\#\#}$, $p < 0.005$; $^{\#}$, $p < 0.05$).

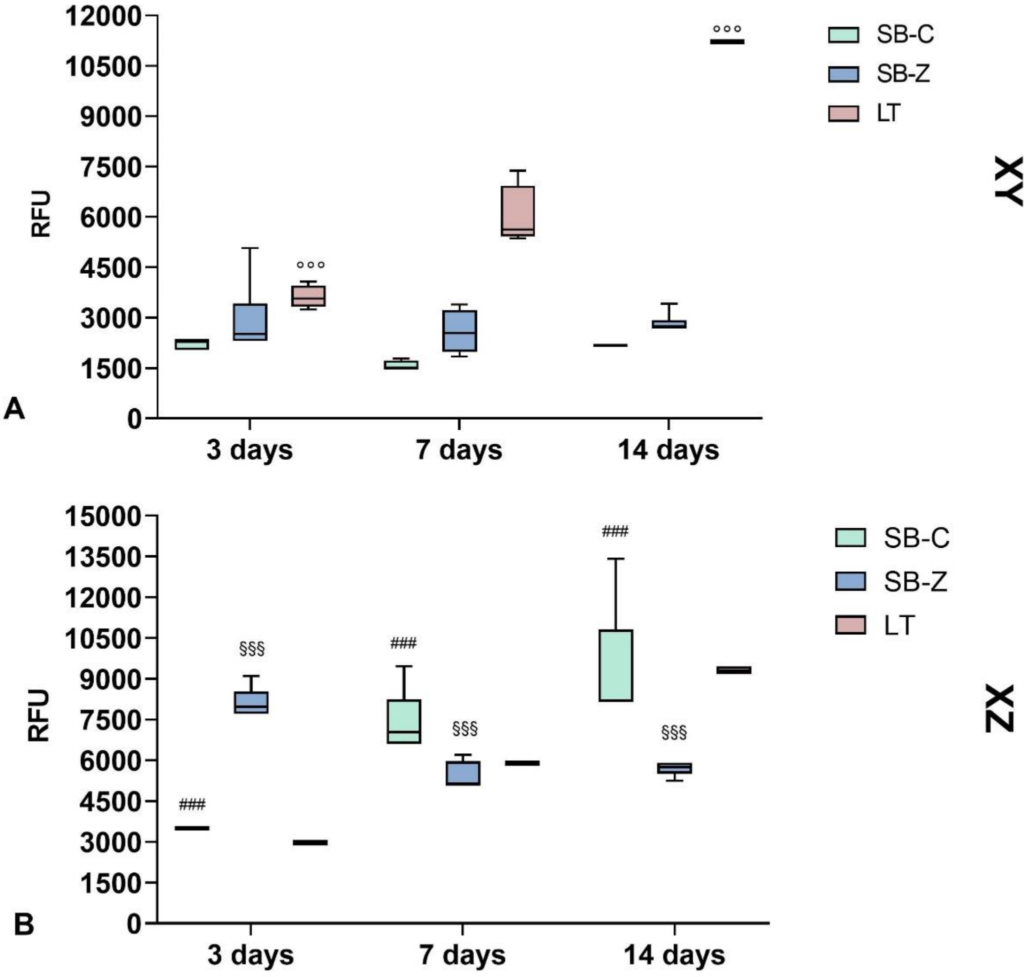

**3 days**	$^{\#\#\#}$ XZ SB-C *vs* XY SB-C	§§§ XZ SB-Z *vs* XY SB-Z	°°° XY LT *vs* XZ LT
**7 days**	$^{\#\#\#}$ XZ SB-C *vs* XY SB-C	§§§ XZ SB-Z *vs* XY SB-Z	
**14 days**	$^{\#\#\#}$ XZ SB-C *vs* XY SB-C	§§§ XZ SB-Z *vs* XY SB-Z	°°° XY LT *vs* XZ LT

<u>PANEL 3</u>. *TGF-β1, BMP-2* gene expression at 7 and 14 days of culture of osteoblasts seeded onto different surfaces finishing conditions realized onto titanium samples with XY orientation (A and C) or XZ orientation (B and D). The results show the mean ± and [95% CI] obtained from three replicate materials.

Statistical analysis is reported in the graphs: between materials with the same types of surface finishing and different orientation at the same experimental time, three different symbols were used: # for SB-C; § for SB-Z and ° for LT (for example $^{\#\#\#}$, $p < 0.001$; $^{\#\#}$, $p < 0.005$; $^{\#}$, $p < 0.05$).

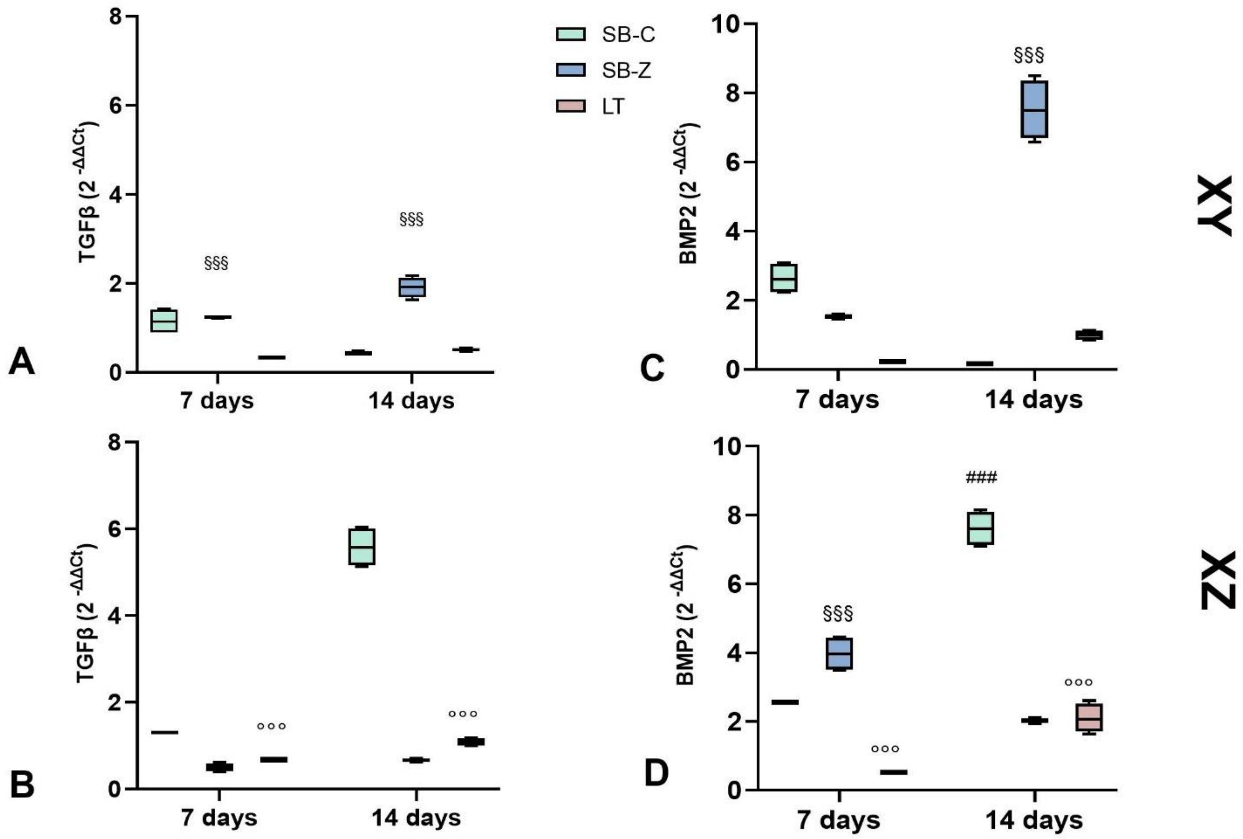

**TGF-β**	7 days	§§§ XY SB-Z *vs* XZ SB-Z	°°° XZ LT *vs* XY LT	
	14 days	§§§ XY SB-Z *vs* XZ SB-Z	°°° XZ LT *vs* XY LT	### XZ SB-C *vs* XY SB-C
**BMP-2**	7 days	§§§ XZ SB-Z *vs* XY SB-Z	°°° XZ LT *vs* XY LT	
	14 days	### XZ SB-C *vs* XY SB-C	§§§ XY SB-Z *vs* XZ SB-Z	°°° XZ LT *vs* XY LT

PANEL 4. ***ALPL*, *COL1A1* and *SSP1* gene expression** at 7 and 14 days of culture of osteoblasts seeded onto different surfaces finishing conditions realized onto titanium samples with XY orientation (A, C and E) or XZ orientation (B, D and F). The results show the mean ± and [95% CI] obtained from three replicate materials.
Statistical analysis is reported in the graphs: between materials with the same types of surface finishing and different orientation at the same experimental time, three different symbols were used: # for SB-C; § for SB-Z and ° for LT (for example ###, $p < 0.001$; ##, $p < 0.005$; #, $p < 0.05$).

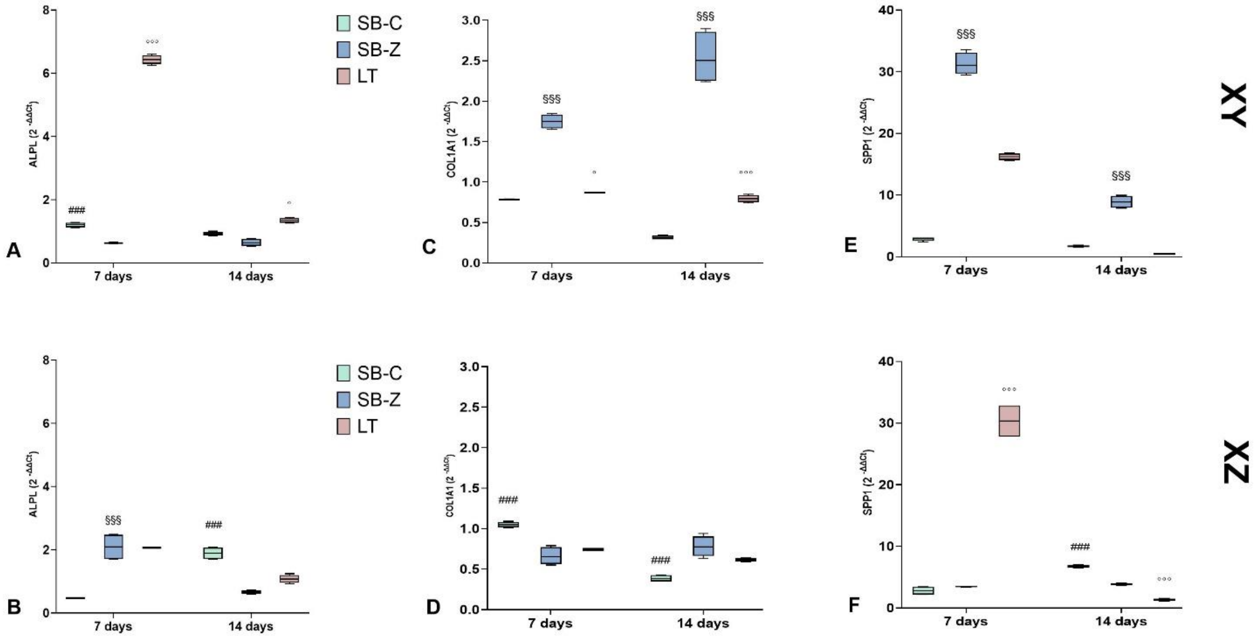

**ALPL**	7 days	### XY SB-C *vs* XZ SB-C	§§§ XY SB-Z *vs* XZ SB-Z	°°° XZ LT *vs* XY LT
	14 days	### XZ SB-C *vs* XY SB-C	° XY LT *vs* XZ LT	
**COL1A1**	7 days	### XZ SB-C *vs* XY SB-C	§§§ XY SB-Z *vs* XZ SB-Z	° XY LT *vs* XZ LT
	14 days	### XZ SB-C *vs* XY SB-C	§§§ XY SB-Z *vs* XZ SB-Z	°°° XY LT *vs* XZ LT
**SSP1**	7 days	§§§ XY SB-Z *vs* XZ SB-Z	°°° XZ LT *vs* XY LT	
	14 days	§§§ XY SB-Z *vs* XZ SB-Z	°°° XZ LT *vs* XY LT	°°° XZ LT *vs* XY LT

PANEL 5. **COL1A1, Osteonectin (SPARC), Osteocalcin** quantitative results of ELISA assays performed onto osteoblasts supernatant in response to XY (A, C and E) or XZ surfaces (B, D and F) sandblasted with corundum (SB-C), zirconia (SB-Z) and laser textured (LT). Data are shown as mean and [95% CI]. Statistical analysis is reported in the graphs: between materialS with the same types of surface finishing and different orientation at the same experimental time, three different symbols were used: # for SB-C; § for SB-Z and ° for LT (for example $^{\#\#\#}$, $p < 0.001$; $^{\#\#}$, $p < 0.005$; $^{\#}$, $p < 0.05$).

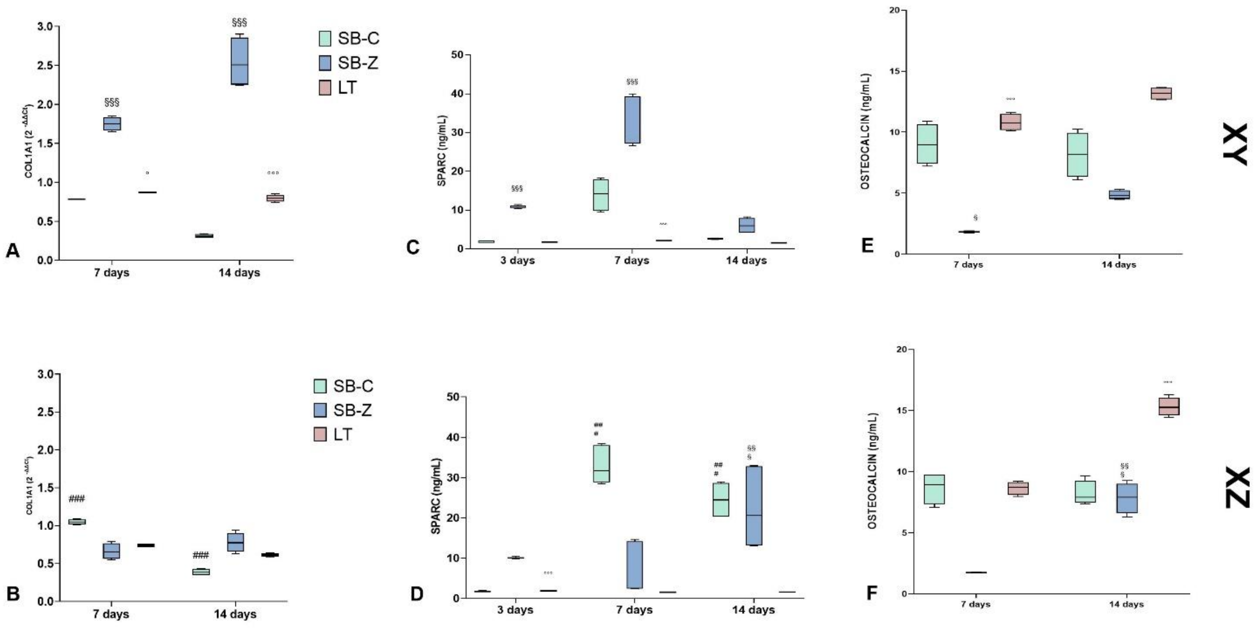

**COL1A1**	3 days	§§§ XY SB-Z *vs* XZ SB-Z	°°° XZ LT *vs* XY LT	
	7 days	### XZ SB-C *vs* XY SB-C	§§§ XY SB-Z *vs* XZ SB-Z	°°° XZ LT *vs* XY LT
	14 days	### XZ SB-C *vs* XY SB-C	§§§ XY SB-Z *vs* XZ SB-Z.	
**SPARC**	3 days	§§§ XY SB-Z *vs* XZ SB-Z	°°° XZ LT *vs* XY LT	
	7 days	### XZ SB-C *vs* XY SB-C	§§§ XY SB-Z *vs* XZ SB-Z	°°° XY LT *vs* XZ LT
	14 days	### XZ SB-C *vs* XY SB-C	§§§ XZ SB-Z *vs* XY SB-Z	
**Osteocalcin**	7 days	§ XY SB-Z *vs* XZ SB-Z	°°° XY LT *vs* XZ LT	
	14 days	§§§ XZ SB-Z *vs* XY SB-Z	°°° XZ LT *vs* XY LT	

PANEL 6. **Interleukin 1β (IL-1β) and Interleukin 6 (IL-6)** quantitative results of ELISA assays performed onto osteoblast's supernatant in response to XY (A and C) or XZ surfaces (B and D) sandblasted with corundum (SB-C), zirconia (SB-Z) and laser textured (LT). Data are shown as mean and [95% CI] . Statistical analysis is reported in the graphs: between materialS with the same types of surface finishing and different orientation at the same experimental time, three different symbols were used: # for SB-C; § for SB-Z and ° for LT (for example $^{###}$, $p < 0.001$; $^{##}$, $p < 0.005$; $^{\#}$, $p < 0.05$).

IL-1β: for all materials the orientation does not statistically significantly influence the production of IL-1β.

IL-6: for all materials the orientation does not statistically significantly influence the production of IL-6.

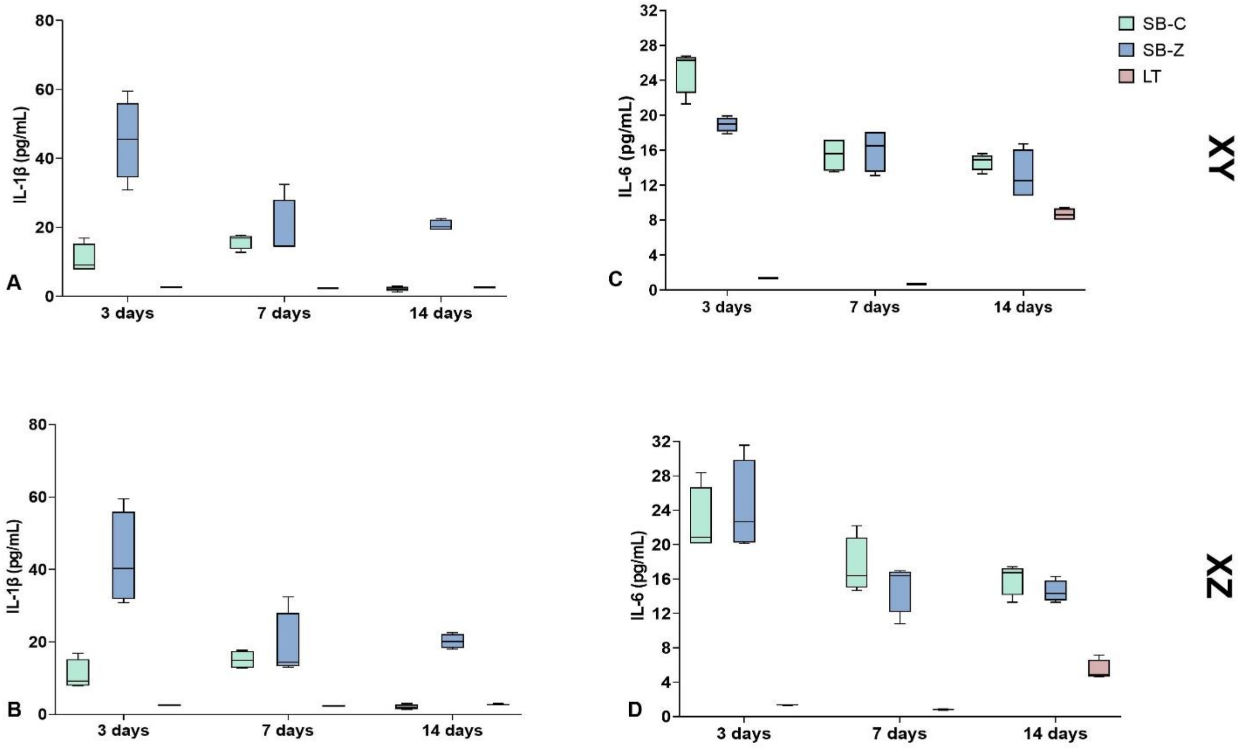

Supplement: Supplementary file 1 — Supporting Information [file ADHM-14-0-s001.pdf]
